# Genetic polymorphisms of *GZMB* and vitiligo: A genetic association study based on Chinese Han population

**DOI:** 10.1038/s41598-018-31233-8

**Published:** 2018-08-29

**Authors:** Meifeng Xu, Yan Liu, Yale Liu, Xiaoli Li, Gang Chen, Wei Dong, Shengxiang Xiao

**Affiliations:** 1 Department of Dermatology, the Second Affiliated Hospital of Xi’an Jiao Tong University, Xi’an, Shaanxi China; 2 Key Laboratory of National Ministry of Health for Forensic Sciences, School of Medicine & Forensics, Xi’an Jiaotong University, Xi’an, China; 3 Department of Laboratory Medicine, the Second Affiliated Hospital of Xi’an Jiao Tong University, Xi’an, Shaanxi China

## Abstract

Vitiligo is a skin disease that affects 1% of the population worldwide. Both environmental and genetic factors contribute to the risk of vitiligo. *GZMB* encodes the enzyme Granzyme B, which plays an important role in cytotoxic T cell-induced apoptosis, and it has been considered one of the candidate genes for vitiligo because of its connections with human immune system. Overall, 3,120 study subjects with Chinese Han ancestry were recruited, and 15 pre-selected SNPs of *GZMB* were genotyped. Genetic association analyses were performed to evaluate the genetic risk of these SNPs to vitiligo. Further bioinformatic analyses were conducted to examine the potential biological function of targeted SNPs. The SNP rs8192917, a non-synonymous coding SNP, was identified to be significantly associated with the disease status of vitiligo, with OR = 1.39 and *P* = 1.92 × 10^−8^. Differences in the association signal can be observed in the stratification analyses of multiple clinical variables. Our positive results provide additional supportive evidence that *GZMB* gene is an important locus for vitiligo in Han Chinese population.

## Introduction

Vitiligo is a skin disease characterized by the loss of pigment in patches of skin^1^. Approximately 1% of the world’s population is affected by vitiligo, and in some countries, this percent can be as high as 2–3%^2^. In general, there is no significant difference in gender for susceptibility to vitiligo. Approximately half of vitiligo patients develop this disorder before 20 years old, and the age of onset of vitiligo for most patients is before 40^1^. Currently, there is no cure for vitiligo but several treatment options can relieve the symptoms^1^. In addition, vitiligo patients may experience depression and relevant mood disorders due to the potential for discrimination from society.

Multiple hypotheses have been proposed for the etiology of vitiligo, and changes in the human immune system are considered to be among the most important causes^1^. Previous studies have shown that vitiligo is a complex disorder that is influenced by both environmental and genetic factors. Multiple genes contributed to the onset of this disease^3,4^; the heritability was approximately 46–72%^5,6^. In recent decades, candidate gene-based association studies have successfully mapped susceptibility for many complex diseases^7–13^. Genome-wide association studies have found multiple loci that contribute to the susceptibility of vitiligo. 48 loci have been reported in Caucasians, and a number more in Han Chinese^14–16^. Despite these findings, these loci explain only approximately 25% of the genetic risk of developing vitiligo. More research is needed to fully unravel the genetic mechanisms of vitiligo^17–19^.

*GZMB* is a protein coding gene that is located at 14q12 and has 5 exons, with a length of 3320 bp. The protein product of *GZMB* is an enzyme (Granzyme B) that plays an important role in the process of apoptosis induced by cytotoxic T cells^20,21^. In a recent GWAS study conducted by Jin *et al*. examining European populations, the SNP rs8192917 in *GZMB* was found to be significantly associated with vitiligo^16^. However, this finding has not been replicated in other populations. In this study, we aimed to investigate the potential association between polymorphisms of *GZMB* and vitiligo using 3,120 study subjects with Chinese Han ancestry. Including rs8192917, a total of 15 SNPs were selected for genotyping. The biological functions of targeted SNPs were examined further by bioinformatic analyses. Our results would provide clues for understanding the roles of *GZMB* in the genetic predisposition of vitiligo.

## Methods

### Study Subjects

In this study, 973 unrelated patients with vitiligo and 2,147 age- and gender-matched unrelated controls were recruited from the dermatological department of the Second Affiliated Hospital of Xi’an Jiaotong University. We only included Han Chinese patients who were born in the local area in an effort to have a genetically homogenous cohort of individuals. None of the patients had been subjected to any therapy in the 6 months prior to sampling. None of the healthy subjects showed any clinical evidence or family history of vitiligo or of any other autoimmune disorder. Vitiligo was clinically characterized in patients as segmental and non-segmental. Segmental vitiligo was diagnosed if the disease followed a dermatomal distribution, which involves one segment of the skin and shows early hair whitening and rapid progression. Active vitiligo was defined as the appearance of new lesions or the enlargement of existing lesions in the 3 months before presentation. Written informed consent was obtained from each subject. This research was performed in accordance with the ethical guidelines of the Declaration of Helsinki (version 2002) and was approved by the Ethics Committee of Xi’an Jiaotong University. The characteristic information of the study subjects is summarized in Table 1. No significant differences in distribution in cases and controls were identified for the age or gender of the study subjects.

**Table 1:** Characteristics information of study subjects.

	Controls (N = 2,147)	Cases (N = 973)	Statistics	*P*
Age, mean ± sd	25.7 ± 8.9	25.7 ± 8.8	t = −0.22	0.8235
Gender (%)				
Male	1,258 (69)	575 (31)		
Female	889 (69)	398 (31)	χ^2^ = 0.05	0.8222
Onset Age (%)				
<20	—	565 (58)		
>=20	—	408 (42)		
Stage (%)				
Active	—	776 (80)		
Stable	—	197 (20)		
Type (%)				
Segmental	—	80 (8)		
Non-Segmental	—	893 (92)		
Family History (%)				
Yes	—	141 (14)		
No	—	832 (86)		
Autoimmune Diseases (%)				
Yes	—	20 (2)		
No	—	953 (98)		

### SNP Selection and Genotyping

SNPs with a minor allele frequency (MAF) >0.01, heterozygosity >0.2 and located within the GZMB gene region were extracted for genotyping based on the 1000 genome CHB data. Overall, 15 SNPs were obtained. Genomic DNA was extracted from peripheral blood leukocytes according to the manufacturer’s protocol (Genomic DNA kit, Axygen Scientific Inc., CA, USA). Genotyping was performed for all SNPs using the MassARRAY platform (Sequenom, San Diego, CA, USA). The genotyping results were generated and processed by using Typer Analyzer software (Sequenom)^22^. The final genotyping call rate for each SNP was greater than 99%, and the overall genotyping call rate was 99.9%. The quality of our genotyping results ensured the reliability of further statistical analyses.

### Statistical analyses

MAFs were calculated and Hardy-Weinberg equilibriums were tested for each SNP. Logistic regressions were performed for each SNP to evaluate their potential contributions to the risk of vitiligo. The potential inflation of signals from single markers caused by population stratification were examined by Q-Q plot and a genomic control was applied when necessary. In addition to these single marker-based analyses, we performed haplotype-based analyses to investigate the combinatorial effects of multiple SNPs. The genetic association software Plink was utilized for logistic model regressions^23^. Haploview was used to construct linkage disequilibrium (LD) structures and haplotype-based analyses^24^. A regional association plot was created by LocusZoom^25^. In general, Bonferroni corrections were applied for multiple comparisons. For single marker-based analyses, the threshold of *P* values was 0.05/15 = 0.003.

### Bioinformatics analyses

Two bioinformatics tools were utilized in this study. SIFT^26^ was used to evaluate the potential biological significance for targeted SNPs. In addition, the effects of targeted SNPs on gene expressions from multiple normal human tissues were examined using the GTEx database^27^. Relevant plots were made using the R project ggplot package^28^.

## Results

**Figure 1:**
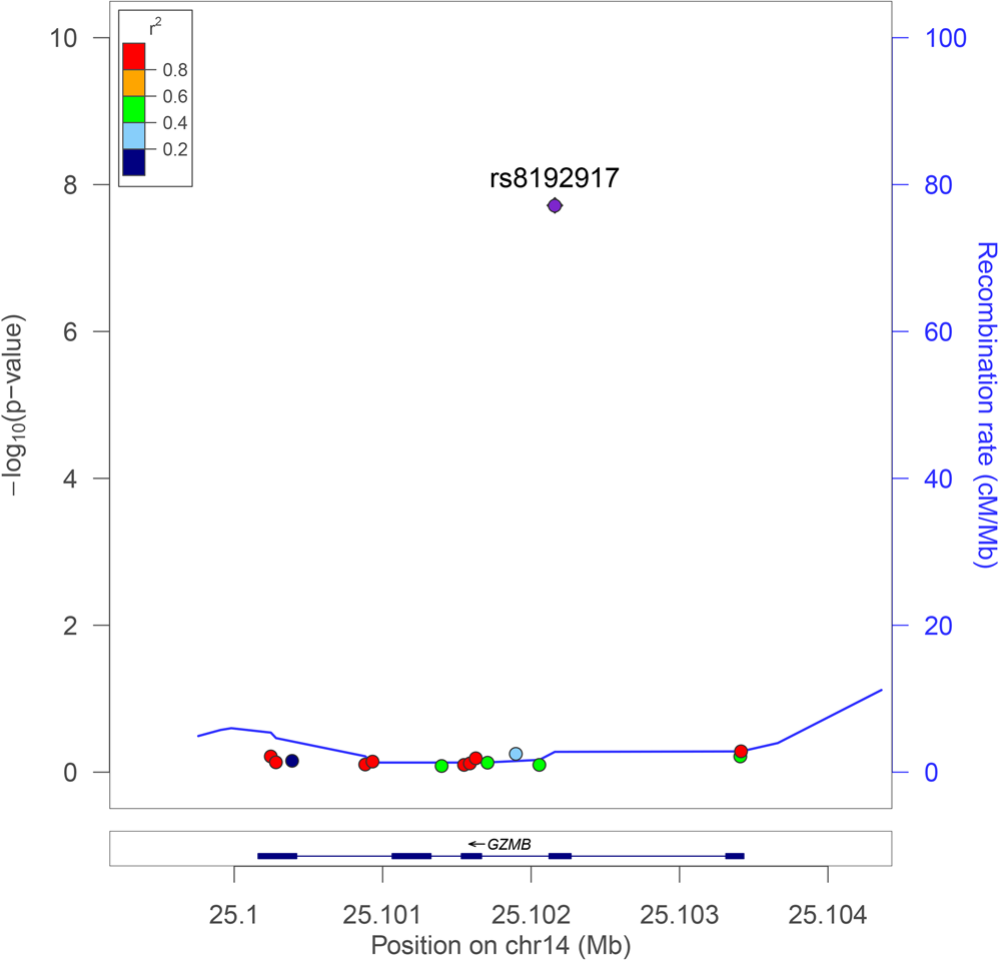
Regional association plot of 15 genotyped SNPs with vitiligo. The most significant SNP (rs8192917) was used as reference to calculate the *r*^2^.

A missense SNP, rs8192917 (Arg55Gln), was identified to be significantly associated with status of vitiligo in our study subjects (Fig. 1). The C allele of this SNP increased the risk of vitiligo by approximately 40% (OR = 1.39, *P* = 1.92 × 10^−8^, Table 2). The significant association signals of this SNP were identified in all three genetic modes, although the additive mode seemed to be most powerful. No other SNP showed significance in single marker-based association analyses. The LD structures constructed using data from the 15 genotyped SNPs are shown in Supplemental Fig. S1. Two 2-SNP LD blocks, including rs2236337-rs2236338, rs6573910-rs6573911, were identified, and no significant LD blocks were found in the haplotype-based analyses (Supplemental Table S1). The Q-Q plot was made based on the results of single marker-based association (Supplemental Fig. S2). No significant inflations of association signals can be identified from this plot.

**Table 2:** Results of single marker based analyses.

CHR	SNP	POS	A1	MAF	HWE	FUNC	OR_ADD	*P*_ADD	OR_DOM	*P*_DOM	OR_REC	*P*_REC
22	rs2236337	24631041	C	0.35	0.89	untranslated-3	0.97	0.608	0.96	0.590	0.97	0.810
22	rs2236338	24631076	G	0.29	1.00	missense	1.02	0.729	1.02	0.777	1.04	0.771
22	rs74345106	24631185	T	0.02	1.00	missense	0.92	0.698	0.92	0.698	NA	NA
22	rs6573910	24631676	T	0.29	0.72	intron	0.98	0.781	0.98	0.844	0.96	0.774
22	rs6573911	24631727	T	0.33	0.77	intron	1.02	0.716	1.02	0.814	1.05	0.689
22	rs71405867	24632191	G	0.17	1.00	intron	1.02	0.816	1.00	0.973	1.20	0.412
22	rs1126639	24632342	A	0.29	0.88	coding-synon	0.98	0.792	0.99	0.866	0.96	0.760
22	rs11539752	24632383	C	0.29	0.60	missense	0.98	0.755	0.98	0.810	0.96	0.772
22	rs10909625	24632423	C	0.29	1.00	coding-synon	1.03	0.647	1.03	0.671	1.04	0.768
22	rs10873219	24632500	T	0.18	0.77	intron	1.02	0.743	1.01	0.866	1.13	0.578
22	rs59268439	24632691	T	0.12	0.84	intron	0.95	0.562	0.97	0.709	0.71	0.346
22	rs9671454	24632850	C	0.04	0.17	intron	0.96	0.787	0.96	0.755	1.10	0.891
**22**	**rs8192917**	**24632954**	**C**	**0.29**	**0.78**	**missense**	**1.39**	**1.92 × 10** ^**−8**^	**1.43**	**3.73 × 10** ^**−6**^	**1.82**	**2.77 × 10** ^**−6**^
22	rs2273843	24634203	C	0.16	0.87	intron	1.04	0.605	1.03	0.767	1.21	0.407
22	rs2273844	24634208	A	0.29	0.92	intron	1.04	0.516	1.05	0.534	1.05	0.703

CHR: chromosome; POS: position of SNPs; A1: tested allele; HWE: *P* values of Hardy-Weinberg Equilibrium; FUNC: functional location of SNP; OR_ADD and *P*_ADD: odds ratio and *P* values for SNP coded as additive mode; OR_DOM and *P*_DOM: odds ratio and *P* values for SNP coded as dominant mode; OR_REC and *P*_REC: odds ratio and *P* values for SNP coded as recessive mode. Significant hit was highlighted in bold.

The eQTL data for rs8192917 extracted from GTEx showed that this SNP was significantly associated with gene expression of *GZMB* in human tibial nerve tissue (*P* = 0.000074, Effect size = 0.28, Supplemental Fig. S3). The result of biological function analyses on rs8192917 using SIFT was “tolerated”, which indicated that this missense SNP would still have a very limited impact on a protein with this mutation.

## Discussion

In this study, we evaluated the genetic association between 15 polymorphisms of *GZMB* and diagnosis with vitiligo based on 3,120 study subjects with Chinese Han ancestry. The results of our single marker-based analyses showed that the C allele of rs8192917indicates an approximately 40% increase in the risk of developing vitiligo in a Chinese population. Compared to an OR of 1.28, as reported by Jin *et al*. in their GWAS meta analyses on European populations^16^, our result was slightly higher, at 1.39. This difference may be due to the different ethnicities of the study subjects. The direct effect of this SNP in both studies was the same. In the European populations, researchers have identified a very high LD pattern among the three non-synonymous SNPs (rs8192917, rs11539752 and rs2236338), resulting in alternative protein haplotypes QPY/RAH^29^. Considering that it is insufficient to draw a reliable conclusion from some SNPs analyses^30–32^, we conducted haplotype analyses and identified a clue for this LD pattern among the three SNPs. However, the LD among these SNPs were not as strong as identified from Europeans. This difference might be due to the difference in population background.

There are several limitations in this study. First, we included only SNPs located within the *GZMB* gene region. However, for most complex disorders, gene expression are often affected by variations located in upstream or downstream regulatory regions (±30 kb) of the targeted gene. Second, the length of *GZMB* is approximately 3,000 bp. Based on data from the 1000 genome project, a rough estimation of the genetic variations in this gene is approximately 300. It is thus impossible to capture all the genetic information of *GZMB*. Furthermore, in order to restrict population stratification we have recruited samples by restricting the subjects with a stable living region^33,34^, but the potential population stratification could not be excluded completely. Therefore, in future studies, DNA sequencing of the upstream and downstream regulatory regions of *GZMB* will be necessary to fully evaluate the genetic contributions to the risk of vitiligo.

In summary, we conducted a candidate gene-based association study to investigate the potential genetic contributions of *GZMB* to the susceptibility of vitiligo. The association signal was identified by single marker-based analyses for a non-synonymous coding SNP rs8192917. Our positive results provide additional supportive evidence that *GZMB* gene is an important locus for vitiligo in Han Chinese population, and are useful for informative assessment of genetic risk for vitiligo in Han Chinese individuals. Given of unknown complex mechanisms in the etiology of vitiligo, followed-up sequencing-based research would be desired in the future to investigate the genetic architecture of the genomic region of *GZMB* and its relationship with vitiligo-related phenotypes.

## Electronic supplementary material

Supplemental materials

## References

[1] Ezzedine K, Eleftheriadou V, Whitton M, Geel NV (2015). Vitiligo. Lancet.

[2] Kruger C, Schallreuter KU (2012). A review of the worldwide prevalence of vitiligo in children/adolescents and adults. International journal of dermatology.

[3] Majumder PP, Das SK, Li CC (1988). A genetical model for vitiligo. American journal of human genetics.

[4] Bhatia PS (1992). Genetic nature of vitiligo. Journal of dermatological science.

[5] Hafez M, Sharaf L, Abd elNabi SM (1983). The genetics of vitiligo. Acta dermato-venereologica.

[6] Das SK, Majumder PP, Majumdar TK, Haldar B (1985). Studies on vitiligo. II. Familial aggregation and genetics. Genetic epidemiology.

[7] Guan F (2012). Association of PDE4B polymorphisms and schizophrenia in Northwestern Han Chinese. Hum Genet..

[8] Guan F (2014). MIR137 gene and target gene CACNA1C of miR-137 contribute to schizophrenia susceptibility in Han Chinese. Schizophr Res..

[9] Chen G, Guan F, Lin H, Li L, Fu D (2015). Genetic analysis of common variants in the HDAC2 gene with schizophrenia susceptibility in Han Chinese. Journal of human genetics..

[10] Guan F (2015). Evaluation of genetic susceptibility of common variants in CACNA1D with schizophrenia in Han Chinese. Scientific reports..

[11] Zhang B (2015). Common variants in SLC1A2 and schizophrenia: Association and cognitive function in patients with schizophrenia and healthy individuals. Schizophr Res..

[12] Guan F (2016). Evaluation of association of common variants in HTR1A and HTR5A with schizophrenia and executive function. Scientific reports..

[13] Guan F (2016). Evaluation of voltage-dependent calcium channel γ gene families identified several novel potential susceptible genes to schizophrenia. Scientific reports..

[14] Jin Y (2010). Variant of TYR and autoimmunity susceptibility loci in generalized vitiligo. The New England journal of medicine.

[15] Jin Y (2012). Genome-wide association analyses identify 13 new susceptibility loci for generalized vitiligo. Nature genetics.

[16] Jin Y (2016). Genome-wide association studies of autoimmune vitiligo identify 23 new risk loci and highlight key pathways and regulatory variants. Nature genetics.

[17] Birlea SA, Gowan K, Fain PR, Spritz RA (2010). Genome-wide association study of generalized vitiligo in an isolated European founder population identifies SMOC2, in close proximity to IDDM8. The Journal of investigative dermatology.

[18] Shen C (2016). Genetic Susceptibility to Vitiligo: GWAS Approaches for Identifying Vitiligo Susceptibility Genes and Loci. Frontiers in genetics.

[19] Spritz RA, Andersen GH (2017). Genetics of Vitiligo. Dermatologic clinics.

[20] Dahl CA (1990). Isolation of a cDNA clone encoding a novel form of granzyme B from human NK cells and mapping to chromosome 14. Hum Genet.

[21] Crosby JL, Bleackley RC, Nadeau JH (1990). A complex of serine protease genes expressed preferentially in cytotoxic T-lymphocytes is closely linked to the T-cell receptor α- and δ-chain genes on mouse chromosome 14. Genomics.

[22] Guan F (2012). Association study of a new schizophrenia susceptibility locus of 10q24.32-33 in a Han Chinese population. Schizophr Res..

[23] Chang CC (2015). Second-generation PLINK: rising to the challenge of larger and richer datasets. Gigascience.

[24] Barrett JC, Fry B, Maller J, Daly MJ (2005). Haploview: analysis and visualization of LD and haplotype maps. Bioinformatics.

[25] Pruim RJ (2010). LocusZoom: regional visualization of genome-wide association scan results. Bioinformatics.

[26] Kumar P, Henikoff S, Ng PC (2009). Predicting the effects of coding non-synonymous variants on protein function using the SIFT algorithm. Nature Protocols.

[27] Lonsdale J (2013). The Genotype-Tissue Expression (GTEx) project. Nature genetics.

[28] Team CR, Team RDC. R (2016). A Language And Environment For Statistical Computing. R Foundation for Statistical Computing: Vienna, Austria. Computing.

[29] Ferrara TM (2013). Risk of generalized vitiligo is associated with the common 55R-94A-247H variant haplotype of GZMB (encoding granzyme B). J Invest Dermatol..

[30] Guan F (2013). A population-based association study of 2q32.3 and 8q21.3 loci with schizophrenia in Han Chinese. Journal of psychiatric research..

[31] Yang H (2013). 4q22.1 contributes to bone mineral density and osteoporosis susceptibility in postmenopausal women of Chinese Han population. PloS one..

[32] Guan F (2016). Two-stage association study to identify the genetic susceptibility of a novel common variant of rs2075290 in ZPR1 to type 2diabetes. Scientific reports..

[33] Guan F (2016). Two-stage replication of previous genome-wide association studies of AS3MT-CNNM2-NT5C2 gene cluster region in a large schizophrenia case-control sample from Han Chinese population. Schizophr Res..

[34] Jia X (2016). Two-stage additional evidence support association of common variants in the HDAC3 with the increasing risk of schizophrenia susceptibility. American journal of medical genetics. Part B, Neuropsychiatric genetics..

