# Supplementary material for: Genetic polymorphisms of GZMB and vitiligo: A genetic association study based on Chinese Han population
Source: Sci Rep. 2018 Aug 29;8:13001. doi: 10.1038/s41598-018-31233-8 (PMC6115438; doi:10.1038/s41598-018-31233-8)
Supplement: Supplementary file 1 — Supplemental materials [file 41598_2018_31233_MOESM1_ESM.docx]

***Title***: Genetic polymorphisms of *GZMB* and vitiligo: A genetic association study based on Chinese Han population

***Author names and affiliations***: Meifeng Xu ^1^, Yan Liu ^1^, Yale Liu ^1^, Xiaoli Li ^1^, Gang Chen ^2^ , Wei Dong ^3^and Shengxiang Xiao ^1^

^1^ Department of Dermatology, the Second Affiliated Hospital of Xi'an Jiao Tong University, Xi'an, Shaanxi, China;

^2^ Key Laboratory of National Ministry of Health for Forensic Sciences, School of Medicine & Forensics, Xi’an Jiaotong University, Xi’an, China;

^3^ Department of Laboratory Medicine, the Second Affiliated Hospital of Xi'an Jiao Tong University, Xi'an, Shaanxi, China

***Corresponding Author***:

Shengxiang Xiao, Department of Dermatology, the Second Affiliated Hospital of Xi’an Jiaotong University, 157 Xiwu Road, Xincheng District, Xi'an, 710004, China.

Tel: 86-29-87679329; Fax: 86-29-87679329; E-mail: shxaxiaoxjtu@163.com

Supplemental Table S1. Results of haplotype based analyses.

| LOCUS | HAPLOTYPE | F_A | F_U | CHISQ | DF | *P* | SNPS |
| --- | --- | --- | --- | --- | --- | --- | --- |
| H1 | OMNIBUS | NA | NA | 0.822 | 2 | 0.663 | rs2236337\|rs2236338 |
| H1 | CG | 0.29 | 0.29 | 3.42×10^-7^ | 1 | 1.000 | rs2236337\|rs2236338 |
| H1 | CA | 0.05 | 0.06 | 0.801 | 1 | 0.371 | rs2236337\|rs2236338 |
| H1 | TA | 0.66 | 0.65 | 0.192 | 1 | 0.661 | rs2236337\|rs2236338 |
| H2 | OMNIBUS | NA | NA | 5.111 | 2 | 0.078 | rs6573910\|rs6573911 |
| H2 | TT | 0.29 | 0.29 | 0.391 | 1 | 0.532 | rs6573910\|rs6573911 |
| H2 | CT | 0.05 | 0.04 | 5.009 | 1 | 0.025 | rs6573910\|rs6573911 |
| H2 | CC | 0.66 | 0.67 | 0.143 | 1 | 0.705 | rs6573910\|rs6573911 |

F_A: frequency in cases; F_U: frequency in controls.

**
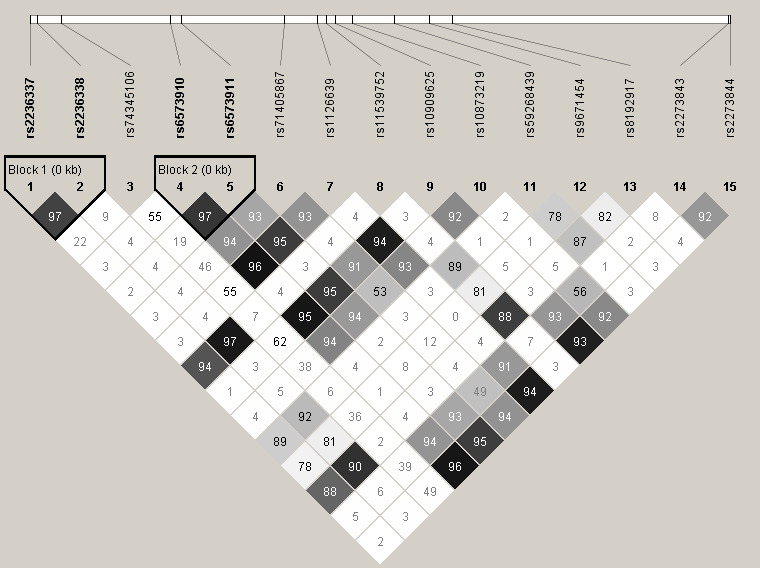
**

Supplemental figure S1. LD structure of 15 SNPs from *GZMB* in our study subjects. Values of D' were indicated in each square.

Supplemental figure S2. Q-Q plot based on results from single marker based association analyses.


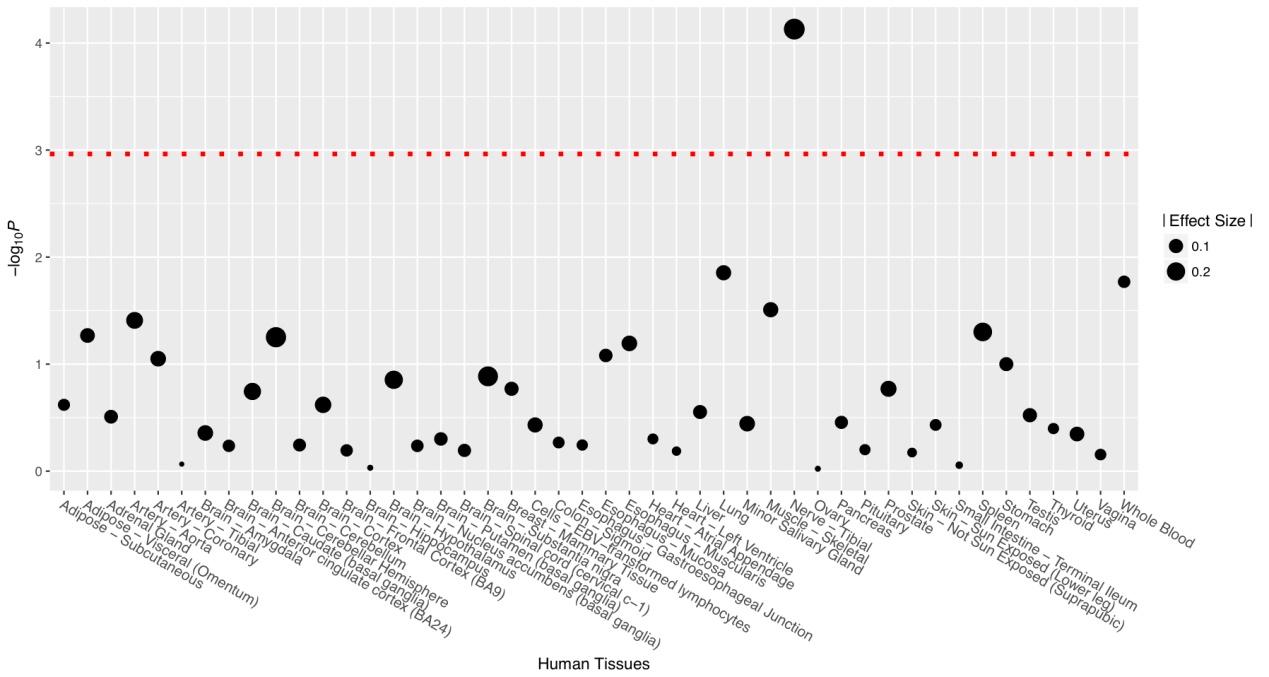


Supplemental figure S3. eQTL data of *GZMB* for rs8192917 in multiple human tissues. Data were extracted from GTEx database. *P* value threshold obtained through bonferroni correction was indicated by red dotted line.
